# Gamma Oscillations, Sensory Stimulation, and Glymphatic Function: Toward User-Friendly Auditory Interventions for Brain Health in Aging and Neurodegeneration

**DOI:** 10.3390/medsci14030398

**Published:** 2026-07-17

**Authors:** Peter Wostyn, Piet Goddaer

**Affiliations:** 1 Department of Psychiatry, PC Sint-Amandus, Reigerlostraat 10, 8730 Beernem, Belgium; 2 Studio Ozark Henry, 8670 Wulpen, Belgium; us@ozarkhenry.com

**Keywords:** 40 Hz auditory stimulation, Alzheimer’s disease, glymphatic system, immersive gamma music, neurodegenerative diseases

## Abstract

The glymphatic system is a brain-wide clearance pathway that facilitates the removal of interstitial solutes, including amyloid-β, and plays a critical role in maintaining brain homeostasis. Impairments in glymphatic transport have been implicated in aging and neurodegenerative diseases, including Alzheimer’s disease. While glymphatic activity is most pronounced during sleep, emerging evidence suggests that specific patterns of neural activity, including gamma-frequency oscillations entrained by sensory stimulation, can modulate glymphatic transport even during wakefulness. Preclinical studies further indicate that 40 Hz sensory stimulation, delivered via light, sound, or multisensory paradigms, can induce gamma oscillations, reduce pathological protein accumulation, and enhance cognitive performance in animal models of Alzheimer’s disease. Early clinical investigations similarly suggest that gamma-frequency sensory stimulation may improve blood-based biomarkers, neuroimaging measures, and cognitive outcomes in patients with Alzheimer’s disease. To translate gamma-frequency stimulation into broadly applicable preventive or therapeutic strategies, approaches must be both effective and tolerable for long-term use. Conventional auditory gamma stimulation can be perceived as acoustically rough or monotonous, reducing listener comfort and limiting acceptability for prolonged use in broader populations. User-friendly auditory formats, such as “gamma music” and the more recently introduced “immersive gamma music”, have been proposed as potentially useful approaches for delivering gamma-frequency stimulation while improving listening comfort and facilitating sustained use. Collectively, gamma-frequency sensory stimulation represents a promising approach to support healthy brain aging and mitigate neurodegenerative processes, particularly when implemented via user-friendly auditory formats that facilitate repeated and long-term use. While these findings are encouraging, further research is needed to validate these approaches and determine their clinical relevance.

## 1. Introduction

Because the brain lacks a conventional lymphatic drainage system, the elimination of metabolic waste long remained an enigma until the discovery of the glymphatic system [1,2]. In 2012, the Nedergaard laboratory first described this brain-wide clearance pathway in the rodent brain [2]. Since then, magnetic resonance imaging studies have provided evidence for the existence of a glymphatic-like clearance pathway in humans [3,4,5]. A growing body of research suggests that impaired glymphatic transport plays a critical role in aging [6] and neurodegenerative diseases, particularly those characterized by the accumulation of misfolded proteins, such as Alzheimer’s disease (AD) [7,8].

Against this background, this review provides an overview of glymphatic system physiology and highlights evidence linking glymphatic dysfunction to aging and neurological disorders. It then summarizes current evidence on the relationship between 40 Hz sensory stimulation and glymphatic transport. Next, recent experimental studies using this stimulation paradigm in AD mouse models and patients with AD are discussed, followed by an outline of emerging user-friendly auditory strategies, including “gamma music” and “immersive gamma music”, that have been proposed as comfortable and well-tolerated approaches for delivering gamma-frequency auditory stimulation.

## 2. The Glymphatic System: Physiology and Dysfunction in Aging and Neurodegeneration

**Figure 1:**
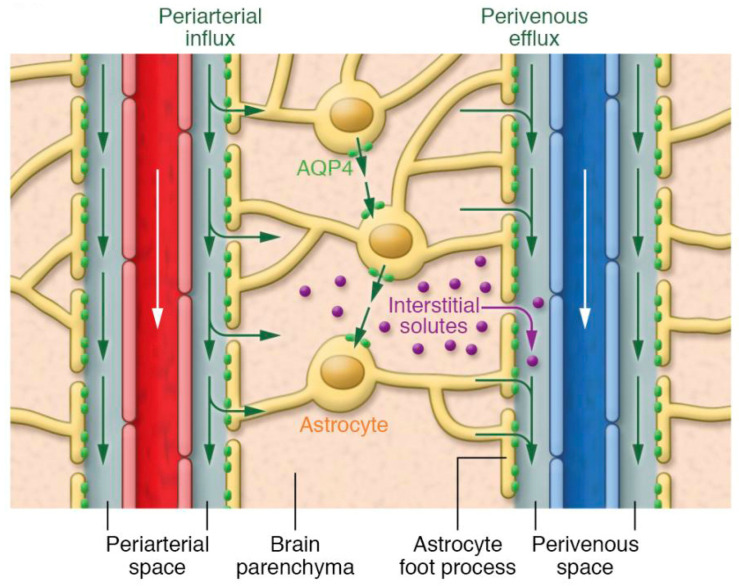
Overview of the glymphatic system. Cerebrospinal fluid enters the brain along periarterial spaces, where it exchanges with interstitial fluid within the brain parenchyma. The resulting fluid and solutes are subsequently cleared along perivenous pathways. This glymphatic transport process is facilitated by aquaporin-4 (AQP4) water channels, which are expressed in a highly polarized manner in the astrocytic endfeet ensheathing the cerebral vasculature. White arrows represent the direction of blood flow, green arrows indicate the movement of cerebrospinal fluid and interstitial fluid, and purple dots denote interstitial solutes. Reproduced from Ray et al. [12] under the terms of the Creative Commons Attribution 4.0 International License.

According to the glymphatic model, cerebrospinal fluid (CSF) enters the brain along periarterial spaces, where it exchanges with interstitial fluid (ISF) within the brain parenchyma [2]. Interstitial solutes, including amyloid-β (Aβ), are then cleared along perivenous pathways and ultimately drained via meningeal lymphatic vessels into the cervical lymph nodes [2,9,10,11]. Glymphatic transport is facilitated by aquaporin-4 (AQP4) water channels, which are highly expressed at the astrocytic endfeet surrounding cerebral blood vessels [11]. An overview of the glymphatic pathway is illustrated in Figure 1 (reproduced from [12]).

Several physiological factors influence glymphatic flow, including arterial pulsatility, respiration, and slow vasomotion [11,13]. Among these, cerebral arterial pulsatility has been proposed as a major propagator of perivascular CSF flow [14]. Glymphatic function is also strongly influenced by brain state: glymphatic activity is largely suppressed during wakefulness and peaks during natural sleep, particularly non-rapid eye movement slow-wave sleep, or under certain anesthetic conditions [15,16,17]. In mice, both natural sleep and ketamine/xylazine anesthesia increase CSF influx by approximately 20-fold and Aβ clearance by twofold compared with the awake state [15]. Additionally, the interstitial volume fraction increases by approximately 60% during natural sleep or ketamine/xylazine anesthesia compared with wakefulness, likely reducing resistance to ISF flux and inward movement of CSF [15]. Norepinephrine, released by the locus coeruleus, suppresses glymphatic activity during wakefulness by contracting the interstitial space [15], whereas electroencephalographic (EEG) slow-wave activity (SWA) appears to promote glymphatic transport [17]. Slow waves, or delta oscillations (0.5–4 Hz), are high-amplitude brain waves characteristic of deep sleep and certain anesthetic states [17]. A recent study comparing EEG activity and CSF tracer influx across six anesthesia conditions in mice found that anesthetics associated with high SWA, such as ketamine/xylazine, produced the highest glymphatic influx [16].

Glymphatic function declines markedly with age [6]. In aging mice, this is associated with reduced cerebral arterial pulsatility and widespread loss of perivascular AQP4 polarization [6]. Glymphatic dysfunction has also been linked to multiple neurological disorders, including AD, Parkinson’s disease, and idiopathic normal pressure hydrocephalus [3,7,8,18,19]. Accordingly, identifying strategies to enhance glymphatic function may be critical for both preventing and treating neurodegenerative diseases and for maintaining overall brain health.

Given that glymphatic activity is strongly reduced during wakefulness, clearance has traditionally been considered largely inaccessible in the awake brain. However, this low baseline activity also suggests a substantial dynamic range for modulation. In this context, emerging evidence indicates that gamma oscillations can influence components of glymphatic transport even during wakefulness [20]. The following section focuses on their potential role in modulating glymphatic function under awake conditions.

## 3. Gamma Oscillations as Modulators of Glymphatic Function

Gamma oscillations (30–100 Hz) are essential for sensory and cognitive processing [21,22], and altered gamma activity has been observed in both AD mouse models and AD patients [23]. Notably, in an AD mouse model, gamma oscillations were reduced early in the disease process, prior to amyloid plaque formation or cognitive decline [22].

Recent experimental evidence suggests that gamma-frequency neural activity in the awake brain can also modulate glymphatic transport [20]. A 2024 study in *Nature* demonstrated that 40 Hz multisensory audiovisual stimulation applied to awake 6-month-old 5xFAD mice increased 40 Hz local field potential power in frontal cortical regions and activated glymphatic transport processes [20]. This was reflected by enhanced CSF influx and ISF efflux, accompanied by reduced amyloid deposition, independent of sleep-state changes [20]. These effects were associated with increased arterial pulsatility, enlargement of meningeal lymphatic vessels, and improved polarization of AQP4 water channels. Vasoactive intestinal peptide interneurons were identified as key mediators of gamma-induced glymphatic modulation through regulation of arterial pulsatility.

Further mechanistic insight into the relationship between gamma-frequency stimulation and glymphatic function has been provided by a recent study demonstrating that 40 Hz light flickering enhances glymphatic influx and efflux in mice, independently of anesthesia and sleep [24]. This effect was linked to adenosine-A_2A_ receptor signaling and was associated with increased astrocytic AQP4 polarization and enhanced vasomotion [24].

In a recent study using a non-human primate model, Wang et al. [25] exposed nine aged rhesus monkeys to 40 Hz auditory stimulation to examine its impact on AD pathology. The monkeys received one hour of 40 Hz auditory stimulation daily for seven consecutive days, resulting in a rapid, twofold increase in CSF Aβ levels. The authors interpreted this increase as consistent with enhanced brain-to-CSF Aβ clearance, potentially involving glymphatic transport mechanisms induced by 40 Hz stimulation. Notably, elevated CSF Aβ levels persisted for more than five weeks after stimulation ended. The authors concluded that their findings provide support for the potential of 40 Hz auditory stimulation as a promising, noninvasive therapeutic approach for AD.

Collectively, the above studies suggest that gamma-frequency activity may influence glymphatic transport processes under awake conditions. By partially overcoming wakefulness-associated suppression of glymphatic function, gamma-frequency sensory stimulation may open the possibility of modulating clearance-related pathways outside of sleep- or anesthesia-dependent states.

Although glymphatic activation represents a compelling mechanistic framework linking gamma-frequency sensory stimulation to enhanced amyloid clearance, other biological pathways have also been proposed to contribute to the reported Aβ-lowering effects of 40 Hz sensory stimulation. These pathways include multiple mechanisms that reduce Aβ production or promote Aβ clearance [26]. On the production side, gamma-frequency light flicker has been shown to activate protein kinase C, which promotes the anchoring of full-length amyloid precursor protein to the plasma membrane and reduces its trafficking to endosomes, thereby limiting β-secretase-mediated cleavage and decreasing Aβ generation [27]. In parallel, several clearance-related mechanisms may contribute to the Aβ-lowering effects of gamma-frequency sensory stimulation. In particular, 40 Hz auditory stimulation has been shown to enhance microglial uptake of Aβ, induce vascular dilation, and promote potential transvascular amyloid transport, collectively supporting Aβ removal through multiple complementary pathways [28]. In addition to modulating Aβ metabolism and removal, gamma-frequency sensory stimulation may exert neuroprotective effects through several other mechanisms, including diminished neuroinflammation, improved synaptic transmission, and upregulated expression of genes associated with synaptic plasticity [29].

In the following sections, we first review preclinical studies examining the effects of gamma-frequency sensory stimulation in animal models of AD, followed by an overview of clinical investigations in patients with AD.

## 4. Preclinical Studies of Gamma-Frequency Sensory Stimulation in Alzheimer’s Disease Mouse Models

In AD mouse models, inducing gamma oscillations through 40 Hz (multi)sensory stimulation has been shown to improve AD-related neuropathology and cognitive decline [22,26,30]. Iaccarino et al. [22] demonstrated that one hour of 40 Hz light flicker induced 40 Hz oscillations and reduced Aβ levels in the primary visual cortex of AD mice. Notably, even in wild-type mice, one hour of 40 Hz light flicker decreased endogenous Aβ levels. Furthermore, daily one-hour sessions of 40 Hz light flicker administered over seven consecutive days significantly reduced amyloid plaque burden in the primary visual cortex of 6-month-old 5xFAD mice.

In a subsequent study, Martorell et al. [28] showed that 40 Hz auditory stimulation induced gamma-frequency neural activity in the auditory cortex and the hippocampal subregion CA1 of 3–8-month-old male wild-type (C57BL/6J) mice. A key outcome of this study was the enhancement of hippocampus-dependent cognitive function, assessed using spatial and recognition memory tasks, in 6-month-old 5xFAD mice following seven consecutive days of one-hour daily 40 Hz auditory stimulation. In these animals, seven consecutive days of one-hour daily 40 Hz auditory stimulation also resulted in reductions in both soluble and insoluble Aβ levels, as well as decreased plaque load in the auditory cortex and hippocampus. The authors further demonstrated that combined auditory and visual stimulation at 40 Hz induced gamma-frequency neural activity in the auditory cortex, hippocampal subregion CA1, and medial prefrontal cortex of 3–8-month-old male wild-type (C57BL/6J) mice. In 6-month-old 5xFAD mice, seven consecutive days of one-hour daily multisensory stimulation reduced soluble and insoluble Aβ levels in these regions and produced a widespread reduction in amyloid plaques throughout the neocortex.

Subsequent studies have likewise reported beneficial effects of 40 Hz sensory stimulation in preclinical AD models [27]. However, not all investigations have reproduced these positive outcomes. For example, Soula et al. [31] found that 40 Hz light flicker failed to engage native gamma oscillations and did not significantly reduce amyloid burden in AD mouse models. Wilson et al. [32] used 40 Hz optogenetic activation of basal forebrain parvalbumin-positive neurons, rather than sensory stimulation, in 5xFAD mice and observed an increase in amyloid load across several brain regions, including the medial prefrontal cortex and septal nuclei. The authors suggested that, because cortical gamma oscillations can be induced through different mechanisms, methodological differences may trigger distinct molecular and cellular responses, which in turn could produce opposing effects on AD pathology [32].

The heterogeneous findings across preclinical studies likely reflect methodological and biological differences rather than fundamentally opposing effects of gamma-frequency stimulation. Variability in stimulation modality (e.g., optogenetic activation versus sensory stimulation), frequency delivery, duration and intensity of exposure, and disease models may contribute to divergent outcomes. In addition, differences in the neuronal circuits targeted by distinct stimulation approaches may lead to qualitatively different network and molecular responses. This may explain why some paradigms reduce amyloid pathology whereas others show neutral or even opposing effects. These considerations highlight the importance of standardizing stimulation protocols and carefully interpreting preclinical findings within their specific experimental context.

## 5. Clinical Investigations of Gamma-Frequency Sensory Stimulation in Humans

Clinical investigations provide preliminary evidence that 40 Hz sensory stimulation may be effective across the AD spectrum, from mild cognitive impairment (MCI) to mild and moderate AD [33]. Specifically, gamma entrainment induced by auditory and visual 40 Hz stimulation has been associated with beneficial effects on AD-related pathology and cognitive symptoms [23,34].

In a single-blinded, randomized, placebo-controlled pilot trial, Chan et al. [34] evaluated safety, compliance, entrainment, and exploratory clinical outcomes in patients with mild probable AD dementia. Participants received daily one-hour sessions of 40 Hz audiovisual sensory stimulation for three months. The authors reported that chronic daily light and sound stimulation was well tolerated, with similarly high compliance in both the control and active groups. Moreover, the intervention effectively induced 40 Hz entrainment. Compared with the control group, participants receiving 40 Hz stimulation showed preliminary associations with reduced ventricular enlargement and hippocampal atrophy, less loss of functional connectivity, improved performance on an associative memory task, and enhanced measures of daily activity rhythmicity after three months of daily stimulation [34], findings that may be consistent with a possible delay in disease progression.

Additional clinical trials have investigated gamma-frequency sensory stimulation in AD patients and have reported encouraging findings, although these studies were limited to relatively short intervention periods ranging from one to six months [35,36,37]. Chan et al. [38] extended these observations by examining the long-term effects of daily one-hour 40 Hz audiovisual stimulation on cognitive outcomes and biomarkers in a small open-label cohort of five patients with mild AD who were followed for approximately two years. At baseline, three participants, all female, were diagnosed with late-onset AD, whereas the remaining two participants, both male, had early-onset AD. The authors reported that prolonged daily 40 Hz audiovisual stimulation was safe and feasible over the two-year period and was associated with a potential slowing of cognitive decline and biomarker progression, particularly among patients with late-onset AD. Notably, these individuals maintained robust EEG-based 40 Hz entrainment, whereas the early-onset AD patients exhibited a marked decline in stimulation-evoked 40 Hz power at approximately 30 months of follow-up. One of the most striking observations was a substantial reduction in plasma concentrations of phosphorylated tau 217 (pTau217), a biomarker closely linked to AD pathology and amyloid burden, in the two late-onset AD patients for whom follow-up blood samples were available. In these individuals, plasma pTau217 levels decreased by 47% and 19.4%, respectively, after approximately two years of daily stimulation, suggesting that long-term 40 Hz audiovisual stimulation may be associated with attenuation of amyloid-related pathological processes.

Although the available clinical studies provide encouraging preliminary evidence regarding the feasibility and potential biological effects of gamma-frequency sensory stimulation, the current evidence base remains limited by small sample sizes, relatively short study durations, heterogeneous study designs, and exploratory endpoints. Larger, well-designed, adequately powered randomized controlled trials are therefore required before firm conclusions can be drawn regarding the prevention of AD, cognitive improvement, or neuroprotective effects.

## 6. User-Friendly “Gamma Music”: An Emerging Auditory Strategy for Gamma-Frequency Stimulation

If gamma-frequency sensory stimulation proves effective as a noninvasive AD treatment method, its potential applications could extend beyond patient-focused therapeutic interventions to preventive strategies aimed at maintaining brain health during aging. In this context, repeated or long-term exposure to 40 Hz auditory stimulation could become relevant not only in clinical settings but also in everyday environments, including home-based use by older adults before the onset of cognitive impairment. Ensuring a comfortable and pleasant form of stimulation would therefore be essential to facilitate sustained adherence and large-scale preventive implementation.

Although recent clinical observations indicate that prolonged daily exposure to 40 Hz audiovisual stimulation is feasible in patients with AD [38], conventional auditory gamma stimulation can be perceived as acoustically rough or monotonous. Such perceptual characteristics may reduce listener comfort and limit acceptability when stimulation is intended for long-term or preventive use in broader populations. To address this limitation, Yokota et al. [39] proposed “gamma music”, an acoustic approach that combines 40 Hz auditory stimulation with musical elements. In their study, gamma music consisted of drums, bass, and keyboard sounds, each engineered to contain a 40 Hz oscillatory component. Notably, gamma music, particularly the gamma keyboard sound, elicited a robust 40 Hz auditory steady-state response while simultaneously providing a relaxed, comfortable, and pleasant listening experience [39].

Qualitative research further reinforces these concerns regarding the user experience of conventional 40 Hz auditory stimulation. In a recent study involving older adults with MCI, Wang et al. [40] explored participants’ subjective experiences with three auditory interventions: self-selected music, 40 Hz sound, and 40 Hz music. The latter consisted of self-selected music combined with 40 Hz sound stimulation. The results revealed that participants favored self-selected music for its memory-boosting and emotional benefits, while roughly half reported negative reactions to the 40 Hz sound, describing feelings of discomfort, irritation, and even torment. By contrast, 40 Hz music elicited a more positive auditory experience, suggesting that this approach may enhance user comfort while reducing the negative aspects of the 40 Hz sound. In addition, the qualitative findings in the study by Wang et al. [40] indicated that participants’ personal connection to music, including its emotional significance, influenced the acceptability and engagement with the 40 Hz music condition. These observations suggest that incorporating musically meaningful or personally relevant auditory content into gamma stimulation paradigms may enhance user engagement and adherence, particularly in populations at risk for cognitive decline.

Together, these findings indicate that gamma music can effectively induce gamma-range neural activity while substantially improving subjective comfort compared with conventional 40 Hz auditory stimulation. Embedding personally significant musical elements may further support user engagement by fostering a sense of personal connection and emotional resonance. This combination of listening comfort and personally significant auditory content may support sustained adherence to repeated or long-term stimulation, which is likely required for both therapeutic and preventive applications. By providing a pleasant and meaningful listening experience, gamma music may enhance user acceptance and adherence, facilitating its use not only in research or clinical settings but also in everyday environments, including potential home-based use by older adults prior to the onset of cognitive decline.

## 7. “Immersive Gamma Music”: A Novel Approach for Supporting Brain Health

Immersive sound refers to three-dimensional audio delivered through speakers or headphones, allowing listeners to perceive sound from all spatial directions [41,42,43]. By creating a realistic auditory environment, immersive sound closely approximates natural listening conditions. Building on the concept of gamma music discussed in Section 6, “immersive gamma music” was introduced in 2025 as an approach that integrates 40 Hz auditory stimuli into a spatially immersive, three-dimensional soundscape [44]. This approach offers the advantage of providing a comfortable and enjoyable listening experience while enhancing perceptual immersion. Notably, immersive gamma music has already been successfully developed by co-author Piet Goddaer, a pioneer in immersive sound and a specialist in creating immersive audio from the ground up. Preliminary observations suggest that this approach is highly effective at gently redirecting listeners’ attention away from potential disturbances by providing a comforting auditory environment [45].

Importantly, a recent study by Masitoh and Suprijanto [46] reported that spatial audio stimuli engage more widespread cortical regions and are associated with increased EEG power across frequency bands compared with standard stereo audio. However, gamma-range activity and neural entrainment were not specifically examined. Consequently, it remains unknown whether immersive gamma music generates more widespread and more robust 40 Hz neural entrainment than conventional gamma music. This question requires further experimental investigation.

Immersive gamma music may also facilitate internally directed attention, as realistic soundscapes have been shown to enhance listeners’ sense of presence and emotional realism [47]. Ratings of presence and emotional reactions increase when listeners experience spatialized versus non-spatialized audio [47], a feature particularly relevant for gamma entrainment, given that attentional state strongly influences the robustness of 40 Hz neural oscillations during sensory stimulation [48]. In a study by Ke et al. [48], participants received 40 Hz auditory stimulation under three distinct attentional conditions: (1) focused attention on the 40 Hz sound, (2) attention directed internally toward personal thoughts or memory, and (3) attention externally distracted by a concurrent podcast overlaid with 40 Hz auditory stimulation. Results showed that 40 Hz oscillatory activity was robust under focused attention, even stronger when attention was internally oriented, and markedly reduced under external distraction. These findings indicate that strategies guiding internal attention may optimize gamma-frequency stimulation, whereas interventions prioritizing entertainment alone may inadvertently introduce sensory competition, weakening entrainment [48]. Immersive gamma music may address these challenges. Because ratings of presence and emotional responses increase with spatialized sound [47], this approach is particularly well suited for designing auditory interventions that leverage personalization, autobiographical associations, and emotional resonance. By promoting an internally directed and coherent attentional state, immersive gamma music may enhance comfort while avoiding the type of sensory competition that could otherwise weaken entrainment. In this way, directing attention toward personal thoughts and memory has the potential to enhance gamma-range neural entrainment without introducing externally driven distractions. Furthermore, the immersive and enjoyable auditory experience may help maintain attention over prolonged or repeated sessions, supporting both user adherence and entrainment robustness.

If immersive gamma music were found to promote broader cortical engagement and stronger gamma-range neural entrainment, it could theoretically influence mechanisms implicated in glymphatic transport during wakefulness. However, this hypothesis remains speculative and requires direct experimental validation. In addition, gamma-frequency sensory stimulation delivered in this immersive format may confer neuroprotective benefits through complementary mechanisms beyond glymphatic modulation. The pleasant and immersive listening experience may improve tolerability, potentially facilitating long-term or repeated use, which is particularly important for real-world applications. Overall, immersive gamma music should currently be regarded as a promising conceptual framework that warrants further experimental and clinical investigation rather than an established therapeutic approach.

Importantly, auditory entrainment is typically more spatially limited than visual or multisensory stimulation, and global gamma-wave entrainment in humans remains difficult to achieve via the auditory pathway. Subcortical and frontal structures are difficult to engage via sound-based stimulation alone, in contrast to murine models, where stimulation is often more direct and may involve invasive approaches. Accordingly, caution is warranted when extrapolating findings from animal studies to noninvasive auditory paradigms in humans. Moreover, species differences in brain anatomy, sleep architecture, CSF dynamics, and glymphatic physiology may further limit the direct translation of findings from murine models to humans.

Finally, the effectiveness of immersive gamma music is likely influenced by several moderating factors, including baseline neural activity, individual differences in auditory processing, and the degree of listener engagement or expectancy [49]. These variables warrant systematic investigation in future studies.

## 8. Conclusions

The discovery of the glymphatic system has fundamentally reshaped our understanding of brain waste clearance. Growing evidence indicates that impaired glymphatic transport is implicated in aging and neurodegenerative diseases, including AD. While glymphatic activity is most pronounced during sleep, emerging research suggests that specific patterns of neural activity can modulate key components of glymphatic function even during wakefulness. In this context, gamma-frequency neural activity entrained by sensory stimulation has attracted increasing attention as a potential modulator of glymphatic transport in the awake brain. Preclinical studies in AD mouse models further demonstrate that 40 Hz sensory stimulation can induce gamma oscillations, reduce amyloid burden, and improve cognitive performance. Early clinical investigations similarly suggest that gamma-frequency sensory stimulation may have beneficial effects on blood-based biomarkers, neuroimaging measures, and cognitive outcomes in patients with AD.

To translate these findings into widely applicable preventive or therapeutic strategies, interventions must be both effective and tolerable for long-term use. User-friendly auditory paradigms, including gamma music and the more recently introduced immersive gamma music, have been proposed as potentially useful approaches for delivering gamma-frequency stimulation while offering a pleasant and engaging listening experience. Collectively, gamma-frequency sensory stimulation represents a promising approach to support healthy brain aging and mitigate neurodegenerative processes, particularly when delivered via user-friendly auditory formats that facilitate repeated and prolonged use. Continued interdisciplinary research will be essential to optimize stimulation protocols, clarify underlying mechanisms, and establish the clinical relevance of these approaches across aging and neurodegenerative disorders.

## 9. Future Directions

Although emerging evidence suggests that 40 Hz sensory stimulation may influence glymphatic transport and other neuroprotective mechanisms, several critical questions remain.

First, it is crucial to establish whether 40 Hz stimulation paradigms can reliably enhance glymphatic activity in the awake human brain and whether such effects translate into meaningful neuroprotective or disease-modifying outcomes. To address these questions, future studies should combine 40 Hz stimulation protocols with EEG measures to quantify gamma entrainment and employ advanced neuroimaging and proxy biomarkers to assess glymphatic activity during wakefulness.

Second, future research should aim to optimize stimulation parameters. Key variables include stimulation frequency, modality (auditory, visual, or multisensory), and the temporal structure and duration of exposure, such as whether continuous or intermittent protocols are most effective. Individual factors, including baseline neural oscillatory activity, auditory processing variability, and attentional engagement, may further influence the effectiveness of gamma entrainment, suggesting that personalized stimulation approaches could be beneficial.

Third, clinical studies with larger cohorts and longer follow-up periods are needed to establish the therapeutic and preventive potential of gamma-frequency sensory stimulation in humans. Randomized controlled trials should assess not only cognitive and clinical outcomes but also relevant biomarkers of neurodegeneration and neuroimaging measures of brain structure and function. Advances in neuroimaging techniques capable of evaluating glymphatic transport in vivo may further facilitate the assessment of these interventions.

Finally, the development of user-friendly stimulation approaches remains a key priority for future research. Strategies such as gamma music and immersive gamma music may be further optimized by incorporating personalized, musically meaningful, or personally relevant auditory content into gamma stimulation paradigms. Such personalization may increase user engagement and adherence, thereby improving the efficacy and supporting sustained use in real-world settings. If validated in future studies, these auditory neuromodulation strategies could provide accessible, noninvasive tools to support brain health.

## Data Availability

No new data were created or analyzed in this study. Data sharing is not applicable to this article.

## Abbreviations

The following abbreviations are used in this manuscript:

Aβ	Amyloid-β
AD	Alzheimer’s disease
AQP4	Aquaporin-4
CSF	Cerebrospinal fluid
EEG	Electroencephalographic
ISF	Interstitial fluid
MCI	Mild cognitive impairment
pTau217	Phosphorylated tau 217
SWA	Slow-wave activity
